# In plants, expression breadth and expression level distinctly and non-linearly correlate with gene structure

**DOI:** 10.1186/1745-6150-4-45

**Published:** 2009-11-21

**Authors:** Hangxing Yang

**Affiliations:** 1 T-Life Research Center, Department of Physics, Fudan University, 220 Handan Road, Shanghai 200433, PR China

## Abstract

**Background:**

Compactness of highly/broadly expressed genes in human has been explained as selection for efficiency, regional mutation biases or genomic design. However, highly expressed genes in flowering plants were shown to be less compact than lowly expressed ones. On the other hand, opposite facts have also been documented that pollen-expressed *Arabidopsis *genes tend to contain shorter introns and highly expressed moss genes are compact. This issue is important because it provides a chance to compare the selectionism and the neutralism views about genome evolution. Furthermore, this issue also helps to understand the fates of introns, from the angle of gene expression.

**Results:**

In this study, I used expression data covering more tissues and employ new analytical methods to reexamine the correlations between gene expression and gene structure for two flowering plants, *Arabidopsis thaliana *and *Oryza sativa*. It is shown that, different aspects of expression pattern correlate with different parts of gene sequences in distinct ways. In detail, expression level is significantly negatively correlated with gene size, especially the size of non-coding regions, whereas expression breadth correlates with non-coding structural parameters positively and with coding region parameters negatively. Furthermore, the relationships between expression level and structural parameters seem to be non-linear, with the extremes of structural parameters possibly scale as power-laws or logrithmic functions of expression levels.

**Conclusion:**

In plants, highly expressed genes are compact, especially in the non-coding regions. Broadly expressed genes tend to contain longer non-coding sequences, which may be necessary for complex regulations. In combination with previous studies about other plants and about animals, some common scenarios about the correlation between gene expression and gene structure begin to emerge. Based on the functional relationships between extreme values of structural characteristics and expression level, an effort was made to evaluate the relative effectiveness of the energy-cost hypothesis and the time-cost hypothesis.

**Reviewers:**

This article was reviewed by Dr. I. King Jordan, Dr. Liran Carmel (nominated by Dr. Eugene V. Koonin) and Dr. Fyodor A. Kondrashov.

## Open peer review

Reviewed by Dr. I. King Jordan, Dr. Liran Carmel (nominated by Dr. Eugene V. Koonin) and Dr. Fyodor A. Kondrashov. For the full reviews, please go to the Reviewers' comments section.

## Background

Highly and broadly expressed genes in metazoans have been reported to be shorter, in either coding, intronic or intergenic regions, than limitedly expressed genes [1-4]. Debates over the evolutionary mechanisms underlying this phenomenon are still ongoing. Some attribute it to the outcome of selection for efficiency, as both transcription and translation are costly in either energy or time [1-3]. Another proposal, termed as 'genomic design', argues that the compactness of housekeeping genes stems from their positioning within the open chramatin, such that less regulatory signals are needed for neucleosome formation [4-6]. In addition, regional mutation bias may also contribute to the compactness of actively transcribed genes, since these genes may experience more deletions [3].

The above speculations not necessarily contradict with each other. Recently, Possoli and collegues found that multispecies conserved sequences, which may be important for the precise regulation and function of host genes, appear to impose strong constraints on the evolution of intron size, confirming and extending the genomic-design model [7]. Interestingly, the authors also observed a reduction in intron size for highly expressed genes, which could not be explained by the influence of multispecies conserved sequences and likely be the result of selection for economy. A double-faceted model was thus constructed to compromise the demand of gaining higher regulatory capacity and the requirements of reducing energetic cost.

Highly expressed genes are not always compact. In the unicellular organism *Saccharomyces cerevisiae*, intron length is positively linked with gene expression [8,9], although protein length shows a negagtive relationship with mRNA/protein abundance [10-12]. Similarly, both intron number and intron density are positively correlated with expression levels for the unicellular green algae *Ostreococcus lucimarinus*, which is the smallest free-living eukaryotes known to date and also contains one of the most compact genomes among all known eukaryotes [13]. For the flowering plants *Arabidopsis *and rice, highly expressed genes were found to be longer than lowly expressed ones in the sense of intron number, average intron length, total intron length, CDS length and untranslated region (UTR) length [14]. All these facts directly contradict with the selection for efficiency model.

The situation in higher plants becomes complicated since *Arabidopsis *genes expressed in pollen have reduced intron sizes compared with genes expressed in sporophytes, supporting the selection for efficiency model and also introducing a role of gametophytic selection on genomic configuration [15]. The research could be confirmed by the finding that highly expressed genes in the moss plant *Physcomitrella patens *contain shorter introns, as mosses experience a dominant haploid gametophytic life phase [16]. In addition, given expression level controlled, housekeeping genes of *Arabidopsis *are less compact than tissue-specific genes, conflicting with the hypothesis of genomic design [17]. Besides, research on the plant *Populus tremula *also witnessed negative relationships between gene expression and protein length or intron numbers [18]. It is unclear to what extent have natural selection, or functional requirements, shaped the genomic configuration for plant genes.

To resolve the above puzzles, the correlations for gene expression pattern and primary genomic structure are re-examined for the flowering plants *Arabidopsis *and rice. The re-examination is based on more complete expression data and using new analytical methods. The results show that different aspects of expression pattern have distinct influences on the evolution of sequence structure. Highly expressed genes are significantly reduced in sequence sizes, especially in the sizes of non-coding regions. In contrast, broadly expressed genes tend to contain longer non-coding sequences, which may be necessary for complex regulations. Furthermore, sequence length seems to correlate with expression level in a non-linear way, suggestive of a possibility that expression level may set upper limits for sequence length, or vice versa. Based on these results, the implications for the evolutionary mechanism of gene sequence structure are discussed.

## Results and Discussion

### Different aspects of expression pattern distinctly correlate with gene structure

In this study, two types of expression data were used. One is from experiments implemented on the Massively Parallel Signature Sequencing (MPSS) platform. This technique quantifies gene expression through counting short unique tags (17-20 bp long) coming from messenger RNA (mRNA) [19]. Based on sequencing technology, this type of mRNA abundance assay could avoid the potential cross-hybridization problem occuring between homologous genes in the microrarry experiments. One possible shortcoming for MPSS technology is that the assay of mRNA transcripts is processed in a somewhat random way, which would cause a type of sampling bias. Hence, parallel analysis was performed using the microarray expression data, to verify that results derived from MPSS data are of real biological meanings. In summary, the MPSS data set used here contain expression profiles for 23,535 *Arabidopsis *genes in 15 tissues and 26,016 rice genes in 18 tissues (see Data and Methods); the microarray data for *Arabidopsis *were from a series of experiments about developmental line [20], which contain expression data for 20,460 protein-coding genes (after filtering, see Data and Methods) in 79 tissues; the microarray data for rice genes were retrieved from the Gene Expression Omnibus database of NCBI by the platform NO. GPL2025, which give expression information for 25,482 protein-coding genes in 35 normal tissues (see Data and Methods).

To examine the relationships between gene expression and gene structure for *Arabidopsis *and rice genes, I firstly calculated Spearman's rank sum correlation coefficients (*ρ*) between various structural parameters and total expression level (*Exp*_*tot*_, the sum of expression quantity of a gene acrosss tissues where the gene is expressed). Spearman's *ρ *was dopted because expression level and most structural parameters don't follow normal distribution.

The results show that, number of introns per gene, average/total intron length per gene and UTR lengths are all significantly positively correlated with *Exp*_*tot *_for *Arabidopsis *genes, which are consistent with Ren et al. [14]. However, transcript length, CDS length and average exon length per gene are significantly negatively correlated with *Exp*_*tot*_, deviating from the observation of Ren et al. [14]. For rice genes, the situation is similar as that for *Arabidospsis *genes, except that transcript length does not significantly correlate with *Exp*_*tot *_and average intron length per gene negatively correlate with *Exp*_*tot*_. Interestingly, for both *Arabidopsis *and rice genes, all non-coding region parameters (i.e. intron number, average and total intron length, 5' and 3' UTR length) are significantly positvely correlated with expression breadth (number of tissues where a gene is expressed), while the two exon-related parameters, i.e. CDS length and average exon length, are both significantly negatively correlated with expression breadth (Table 1). Hence, in plants, the proportion of non-coding sequences tend to be higher for broadly than for narrowly expressed genes.

**Table 1:** The correlations between sequence structural parameters and expression pattern for *Arabidopsis *and rice genes.

	*Arabidopsis*			Rice		
		
Parameters	*Exp*_*tot*_	*Exp*_*avg*_	*Width*	*Exp*_*tot*_	*Exp*_*avg*_	*Width*
Length of primary transcript	-0.073	-0.253	-0.001*	-0.002*	-0.226	0.085
		**-0.295**	**0.158**	**-0.291**	**0.202**	
Length of CDS	-0.222	-0.314	-0.167	-0.119	-0.165	-0.086
		**-0.274**	-0.012**		**-0.143**	-0.011*
Average exon length	-0.139	-0.048***	-0.160	-0.033***	0.024***	-0.061
		0.038***	**-0.165**		**0.068**	**-0.104**
Average intron length	0.154	0.037**	0.178	-0.043***	-0.091	-0.007*
		**-0.048**	**0.171**		**-0.115**	**0.075**
Number of introns	0.064	-0.116	0.120	0.054	-0.101	0.101
		**-0.200**	**0.201**		**-0.151**	**0.134**
Intron density	0.151	0.028	0.188	0.144	0.007	0.175
		**-0.083**	**0.226**	**-0.070**	**0.161**	
Total intron length	0.094	-0.093	0.150	0.017**	-0.151	0.080
		**-0.190**	**0.221**		**-0.209**	**0.165**
5' UTR length	0.251	-0.012***	0.297	0.010*	-0.109	0.066
		**-0.133**	**0.280**		**-0.156**	**0.125**
3' UTR length	0.304	0.067***	0.346	0.048***	-0.097	0.104
		**-0.099**	**0.316**		**-0.157**	**0.151**
5' intergenic length	-0.027**	0.022**	-0.054	0.022**	0.018**	0.020**
		**0.075**	**-0.116**		0.014**	0.003*
3' intergenic length	-0.045	0.044	-0.076	-0.047	0.012*	-0.061
		**0.094**	**-0.116**		0.037***	**-0.058**

For each structural parameter, the first line represents the Spearman's rank sum correlations with expression pattern, whereas the second line represents Spearman's partial correlations. Controlled variable for the columns of *Exp*_*avg *_is expression breadth, whereas that for the columns of *Width *is *Exp*_*avg*_. Intron density was defined as the ratio of intron number to CDS length, i.e. intron number per coding base. *Exp*_*tot*_, total expression level; *Exp*_*avg*_, average expression level; *Width*, expression breadth. CDS, Coding Sequence; UTR, Untranslated Region. Significance of correlations: no asterisks, *P *< 1*e *- 10; ***, 1*e *- 10 <*P *< 1*e *- 3; **, 0.001 <*P *< 0.05; *, *P *> 0.05. Numbers in bold indicate highly significant partial correlations (*P *< 1*e *- 10).

Total expression level has been found to be strongly correlated with expression breadth [4]. For *Arabidopsis *genes, Spearman's *ρ *between *Exp*_*tot *_and expression breadth is 0.93, and that for rice genes is 0.94. Consequently, the influences of total expression level on gene structure may be strongly confouneded with that of expression breadth. Another expression parameter, average expression level (*Exp*_*avg*_, average expression quantity of a gene across tissues in which the gene is expressed), shows a weaker correlation with expression breadth (Spearman's *ρ *= 0.70 or 0.81, for *Arabidopsis *or rice genes). Hence, the correlation between structural parameters and *Exp*_*avg *_were examined, in order to separate the effects of expression breadth.

It was found that, for both plants, parameters positively correlated with *Exp*_*tot *_show weaker correlations with *Exp*_*avg *_whereas parameters negatively correlated with *Exp*_*tot *_show stronger correlations with *Exp*_*avg *_(Table 1). The only exception is average exon length per gene, which is more weakly neagtively correlated with *Exp*_*avg *_for *Arabidopsis *genes, or significantly positively correlated with *Exp*_*avg *_for rice genes.

For a further demonstration, I calculated Spearman's partial correlation coefficients between *Exp*_*avg *_and structural parameters, with expression breadth controlled. After the effects of expression breadth have been factored out, all non-coding region parameters are more strongly negatively related with *Exp*_*avg *_(Table 1). Partial correlation of CDS length and *Exp*_*avg *_is a little weaker than full correlation, but remains to be negative and significant. In contrast, partial correlation of average exon length and *Exp*_*avg *_changes to be positive and significant, indicating that the negativeness of the partial correlation for CDS length is in fact determined by the significant negative correlation between intron number (i.e. exon number) and *Exp*_*avg *_(Table 1). Conversely, if the effects of *Exp*_*avg *_are controlled, the positive correlations with expression breadth for non-coding parameters become stronger (or remain essentially the same), corroborating the positive role of expression breadth in determinging the size variation of non-coding sequences for plant genes. The negative correlations with expression breadth for average exon length tend to be stronger, but the residual correlation for CDS length becomes weaker, likely due to the positive association between intron number and expression breadth. Overally, after the effect of *Exp*_*avg *_has been factored out, primary transcript length of plant genes is significantly positively correlated with expression breadth.

All the above analyses were repeated using microarray expression data and essentially the same scenarios could be obtained (see Table S1 in Additional File 1). Moreover, the nature embedded in the data has also been explored in another analyzing scheme, namely principal component analysis (PCA). Shown in Fig. S1 (see Additional File 2), the results of PCA coincide with that of correlation analysis very well. As a result, the above observations are more likely actual biological facts, rather than artifacts generated by technical shortcomings.

In brief, different aspects of expression pattern may affect the evolution of different parts of gene sequences in distinct ways. At one side, gene expression level or cellular mRNA abundance tend to be negatively correlated with structural parameters, especially non-coding region parameters, meaning that highly expressed plant genes tend to be more compact. At the other side, broadly expressed genes tend to contain higher proportion of non-coding sequences, deriving an overall positive relationship between expression breadth and transcript length. Recently, Camiolo et al. obtained the same scenarios for *Arabidopsis *genes using multiple regression analysis [21]. Here, it is confirmed that, this scenario is true for monocots as well as for dicots.

### The non-linear relationship between expression level and sequence length

Although most genic parameters significantly correlate with expression pattern, the absolute values of these correlations are moderate or even very small. The largest observed correlations are that of CDS length and primary transcript length with average expression level and that of UTR lengths with expression breadth, at the level of about 0.3. Correlations of other structural parameters with expression pattern are mostly at the level of 0.2 or 0.1, implying that only a few percentage of the variation in gene sequence length could be explained by expression pattern. One possible reason for these scenarios may be that, gene expression data at the current stage contain a substantial level of noise, which if distributed randomly would significantly weaken the observable correlations.

**Figure 1:**
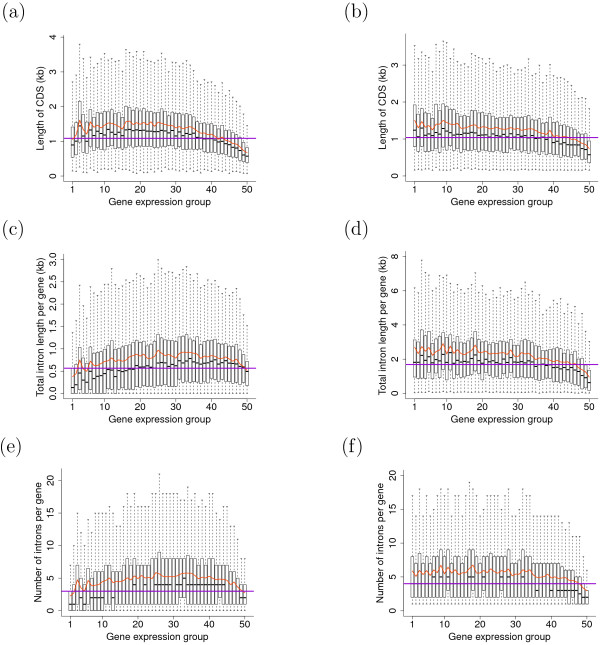
**Boxplots of structural characteristics versus expression level for *Arabidopsis *and rice genes**. In each graph, x-axis represents gene-expression level, boxes represent the range of parameters for each gene group, with bold central lines represent the medians, lower and upper boundaries represent the first and third quartiles respectively, whereas whiskers extend to the most extreme points within 1.5 × interquartile range from the boxes. The red curves represent mean values of parameters for each expression group, whereas horizontal darkviolet lines indicate the population median for each structural parameter. Presented parameters are: CDS length in (a) *Arabidopsis *and (b) rice; total intron length per gene in (c) *Arabidopsis *and (d) rice; number of introns per gene in (e) *Arabidopsis *and (f) rice. Differences in structural parameters between different expression groups are statistically significant (all Kruskal-Wallis rank sum test *P *< 1e-50).

Another possible reason is that, there may not exist simple linear relationships between gene expression pattern and sequence length. To check this point, genes for each plant were sorted into equal-sized groups according to expression level and the distribution of structural parameters for different groups were compared. Indeed, across gene expression groups, median/mean intron number per gene and CDS length curvelinearlly correlate with expression level ranks, whereas median/mean values of average intron length per gene positively correlate with expression level ranks. Furthermore, substantial variations in structural parameters were observed for each expression level group (Figure 1, results based on MPSS expression data; Fig. S2 in Additional File 3, results based on microarray expression data). Genes of each expression breadth group also vary significantly in structural parameters, consistent with the low correlation coefficients observed for gene structure and expression breadth (Fig. S3 in Additional File 4). Taken together, these non-linear correlations between gene expression and gene structure indicate that gene expression could only partly determine the variation of sequence length.

**Figure 2:**
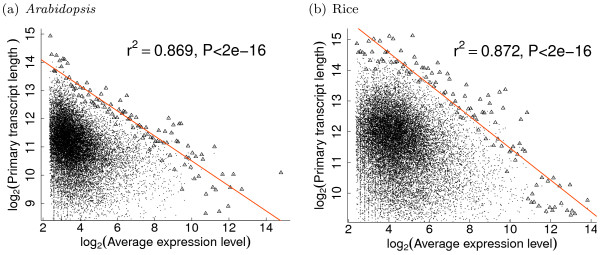
**Extreme values of transcript lengths for plant genes scale as power-laws of expression levels**. In each graph, each point represents one gene in the whole dataset, whereas triangles denote the data subset used to fit the linear line. Axes are all on the logarithmic scale. Expression data were taken from MPSS experiments [19].

However, the extreme values of structural parameters seem to be strongly negatively correlated with expression level. Using expression measures from MPSS experiments, log-transformed extreme structural values were found to be well linearly related with log-transformed expression levels, suggesting that extreme strucural parameters could scale as power-laws of expression levels (see Figure 2 and Table 2). These power-laws are true for various structural characteristics versus either total, average or peak expression levels. In most cases, over 80% of the variations in the extreme structural values could be explained by expression level (Table 2). Previously, Jansen and Gerstein also reported that protein length of yeast genes could scale as a power-law of expression level [22].

**Table 2:** Extreme values of structural parameters scale as power-laws of expression levels for plant genes.

	*Arabidopsis*			Rice		
	
Parameters	*Exp*_*tot*_	*Exp*_*avg*_	*Peak*	*Exp*_*tot*_	*Exp*_*avg*_	*Peak*
Primary transcript length	-0.32(0.88)	-0.44(0.87)	-0.35(0.84)	-0.55(0.82)	-0.52(0.87)	-0.50(0.82)
Intron number per gene	-0.36(0.84)	-0.52(0.84)	-0.34(0.79)	-0.59(0.73)	-0.51(0.74)	-0.63(0.77)
Total intron length	-0.36(0.85)	-0.51(0.86)	-0.39(0.77)	-0.64(0.72)	-0.60(0.78)	-0.76(0.78)
Average intron length	-0.23(0.66)	-0.36(0.67)	-0.22(0.42)	-0.58(0.70)	-0.56(0.79)	-0.66(0.77)
CDS length	-0.33(0.86)	-0.45(0.83)	-0.36(0.80)	-0.51(0.81)	-0.44(0.81)	-0.50(0.80)

Exponents of the power-laws are shown. For each combination of structural and expression variables, the linear regression was done as follows: both variables were firstly log-transformed; the range of expression variable was then equally divided into ~100 spaces; for each space, the median expression value and the maximum structural value were selected; these maximum dependent values were fitted against the medium independent values. Numbers in parentheses show the coeffiecients of determination (*r*^2^) for the linear regressions. For all regressions, *P*-value < 2e-16 according to analysis of variance. *Exp*_*tot*_, total expression level; *Exp*_*avg*_, average expression level; *Peak*, peak expression level across tissues.

The pictures based on microarray expression data seem to be different, as extremes of structural characteristics better scale as logrithmic functions of expression levels. Power-laws could only be observed for *Arabidopsis *genes for extreme structural characteristics versus average expression level (Fig. S4 in Additional File 5). Compared with power-law, the logarithmic function implies higher constraints on sequence length for the most highly expressed genes. Whatever case is true, it seems that in plants expression level may set upper limits for gene sequence length, or vice versa.

### Relevance with previous studies about plant genes

How to relate the facts presented here to previous observations? The fact that CDS length negatively and significantly correlates with expression level is accordant with the observation in *Populus tremula*, although an even stronger negative correlation between CDS length and expression breadth was observed [18]. The fact that intron number and average/total intron length per gene are significantly negatively correlated with expression level is consistent with the observation in the moss plant *Physcomitrella pates*, although the author in that study took the output of EST experiments as gene expression information [16]. Besides, it has been known that genes expressed in pollen tend to have shorter introns than genes expressed in sporophyte tissues which has been intepreted as an evidence supporting gametophytic selection [15]. Gametophytic selection, however, may not be responsible for the results obtained here, since the MPSS expression data for *Arabidopsis *were all from sporophyte tissues (see Data and Methods). In conclusion, the results presented here are congruent with most of the published observations.

However, the results presented here are inconsistent with that from Ren et al., which demonstrated that highly expressed genes in *Arabidopsis *and rice are more compact [14]. To investigate the correlations between structural characteristics and expression level, the authors in that study sorted all genes into equal-sized groups according to expression level. Genes from the top and bottom N% quantiles (where N = 1, 5, 10, etc.) were compared for average strucutural parameters. It was shown that, genes belonging to the higher expressed quantiles are longer than that belonging to the lower expressed quantiles in most aspects of gene structure. Despite the ability to uncover some facts, this analysis may be problematic in two ways. First, it would miss the global view on the relationship between structural characteristics and expression level. On the global scale, most aspects of gene structure negatively correlate with expression level (Table 1 and Table S1). Second, it didn't separate the effects of expression breadth from expression level. Most likely, the observation of Ren et al. reflected the positive correlations of expression breadth versus sequence length, whereas expression levels tend to negatively correlate with sequence length (especially non-coding region length).

### Broadly expressed genes tend to be longer may be common between plants and animals

In mammals, broadly expressed genes, especially housekeeping genes, were found to be more compact than narrowly expressed genes [2,4]. In further examinations, researchers found that mammalian genes that are expressed in a moderate number of tissues on average have the longest sequences, while both narrowly and broadly expressed genes are relatively shorter [23,24]. The curvelinear relationships between gene structure characterisics and expression breadth have been taken as evidence supporting the conjecture of "goldden middle", stating that intermediately expressed genes are sorrounded by longest noncoding sequences because of complex regulations [24]. For plant genes, the curvelinear relationships with expression breadth could be observed for total number of introns per gene and total intron length per gene (see Additional file 4). The curvelinear trends, however, are very weak, suggestive of limited applicability of the "golden middle" principle in plant genomes.

On the other hand, when expression level is controlled, housekeeping genes are in fact not more compact than tissue-specific genes [17], consistent with results presented here. A recent research also found that human housekeeping genes are longer than tissue-specific genes, when many lowly but constitutively expressed genes had additionally been identified as housekeeping genes [25]. That narrowly expressed genes are shorter than broadly expressed genes seems to be common to plant and metazoan species.

For plant genes, the positiveness between gene structure and expression breadth is most pronounced for non-coding regions. Non-coding regions tend to harbor plenty of functional signals necessary for precise regulation of nearby genes [6,26-30].

Higher proportion of non-coding sequences likely reflect the requirements for complex regulations. Therefore, the significant correlations of expression breadth with gene structure support the view that functional requirements contribute to genomic configuration in plants.

### Possible explanations for the shortness of highly expressed genes

Another common trend across plant and metazoan species is the compactness of highly expressed genes, especially in the non-coding regions. This trend seems to be confined to multicellular eukaryotes. Research about unicellular eukaryotes to date uncover reversed scenarios that intron number and/or intron length tend to positively correlate with expression level. This phenomenon is common for the budding yeast *Saccharomyces cerevisiae *and the smallest free-living eukaryotes known to date, the green algae *Ostreococcus lucimarinus*, which also carry one of the most compact genomes among eukaryotes. The distinctions between unicellular and multicellular organisms are intriguing and worthy of further investigation.

Possible explanations for the shortness of highly expressed genes in multicellular eukaryotes include local mutation biases and selection for efficiency [1-3]. The mutational bias model hypothesizes that highly expressed genes tend to position within genomic regions with higher deletion/insertion biases [3]. Local mutation bias would influence the intergenic as well as genic regions, causing intergenic and intragenic non-coding region sizes to vary consistently with expression level. However, for both plants, intergenic length are weakly, but significantly, positively correlated with expression levels, contrasting to the negative correlations between intron sequence length and expression level (Table 1 and Table S1). Consequently, the shortness of highly expressed genes could not be ascribed to local mutation bias. The recent study of Camiolo et al. also reached this conclusion by considering the effects of intergenic length and intergenic GC content simultaneously in a multiple regression framework [21].

The selection for efficiency model hypothesizes that natural selection favors to enhance expression efficiency by means of deletion of functionally neutral sites [1-3]. It encompasses two distinct facets, i.e. the energy-cost hypothesis and the time-cost hypothesis. Both hypotheses are reasonable, as transcription and translation processes are cost in both time and energy [1]. However, the energy-cost hypothesis could not be reconciled with the fact that mammalian genes expressed in large organs are not more compact than genes expressed in small organs, given expression level controlled [31]. On the other hand, evidences supporting the time-cost hypothesis have been accumulated. These include, for example, antisense genes possibly involved in the regulation of corresponding sense genes in human genome tend to contain shorter introns [32,33], eukaryotic genes responded to stress signals or involved in the processes of cell differentiation tend to contain lower number/density of introns [34]. Notably, the time-cost hypothesis actually states that rapidly expressed genes should be compact, rather than highly expressed genes should be compact. For this hypothesis to be able to explain the shortness of highly expressed genes, it must be true that highly expressed genes are generally required to be transcribed/translated with higher rates than lowly expressed genes. Further test and validation are therefore required for either hypothesis to be undoutedly accepted or rejected.

To test the effectiveness of the energy-cost hypothesis, it seems to be helpful to figure out how the energy-cost for gene expression would change with expression level. Energy costs of transcribing one gene come from the syntheses of ribonucleotides--the building blocks of mRNA molecules, and from the polymerization of these building blocks to form the mRNA strand. Suppose the length of primary transcript of one gene is *L*, and the number of transcribed mRNA molecules is *N*. The energy cost (*E*) for the transcription could be estimated to be proportional to the product of L and N, namely *E *∝ L*N. This estimation is conservative, since the removal of spliceosomal introns from primary mRNA also requires energy. But this could not be a problem, as intron number and transcript length consistently vary with expression level. Similarly, the energy cost for translation could be proportional to the product of CDS length and protein expression level.

As genes expressed at the same level vary considerably in sequence length, energy-cost for the expression of genes with similar expression levels would also vary significantly, due to factors other than expression quantity. Therefore, differences in energy-cost between genes with different expression levels may be most pronounced in extreme values, just as the cases of gene structure.

According to gene expression measures from MPSS experiments, extreme transcript lengths (or total/average intron lengths, number of introns per gene) scale as power-laws of expression level (Figure 2 and Table 2), which could be expressed as: *L*_*max *_∝ *N*^-*α*^, where 0 <*α *< 1 is the exponent of power-law and *L*_*max *_represents the maximum transcript lengths for genes expressed at each level. Thus, the extreme energy-cost of transcription could scale as a positive power-law of expression level, i.e. *E*_*max *_∝ *N*^1-*α *^. In other words, the extreme energy-cost for the transcription of plant genes would increase with expression level, suggesting that energetic constraint (if this really happen) for transcription decreases with expression level. Similar trend could be deduced for energy-cost of translational processes, as extreme CDS lengths also scale as power-laws of expression levels. Therefore, the reduction in sequence lengths for highly expressed genes isn't accompanied by reductions in energy-cost for either transcription or translation processes, contradicting with the assumption of energy-cost hypothesis that natural selection acts to shape gene structure in order to minimize energy cost.

According to the expression measures from microarray experiments, extreme sequence lengths (either primary transcript length, total/average intron length per gene or CDS length) most likely scale as logarithmic functions of expression levels which could be expressed as *L*_*max *_= *L*_0_- k * log_2 _*N*, where *L*_0 _> 0 denotes the maximum sequence length when *N *= 1 and *k *> 0 is the scaling factor. The extreme energy-cost *E*_*max *_could then be expressed as *E*_*max *_∝ N * (*L*_0_- k * log_2 _*N*), which has a curvelinear shape (see Additional file 5). In other words, the extreme energy cost would generally increase with expression level, but goes downward for a small fraction of genes that are most highly expressed (< 1.4%, points at the right side of the black dotted lines in Fig. S4, see Additional File 5). Only for these most highly expressed genes, would energy-cost and sequence length consistently decrease with expression level, in accordance with the assumption of energy-cost hypothesis. Therefore, energy-cost couldn't have been the factor dominating the evolution of gene structure on the genomic-scale.

In contrast to energy-cost hypothesis, the time-cost hypothesis could be better reconciled with current data. Previous research found that, multiple polymerases could simultaneously bind to a transcribing mRNA strand [35], causing the time-cost of transcription relates with the number of transcribed mRNA through a sublinear function. In this way, the time-cost for transcribing mRNA could be expressed as *T *∝ L *N^*β*^, where *T *represnts time-cost, *L *denotes the length of transcript, *N *is the number of transcripts, 0 <*β *< 1 is the scaling factor. Hence, either *T *∝ *N*^*β*-*α *^according to the MPSS expression data, or *T *∝ *N*^β ^* (*L*_0_- k * log_2 _*N*) according to the microarray data. In the former case, if *β *>*α*, the extreme time-cost will increase with expression level, but with a lower rate than energy-cost would increase; if *β *= *α*, the extreme time-cost is constant for all expression levels; if *β *<*α*, the extreme time-cost will decrease with expression level, implying higer efficiency requirements for the transcription of highly expressed genes. In the latter case, the proportion of genes that is limited in time-cost for transcription will increase when the scaling factor *β *varies from 1 to smaller values, again implying a higher efficiency requirement for highly expressed genes (Fig. S5 in Additional File 6). Similar conclusions could be derived for translational processes, since multiple ribosomes could simultaneously bind to single mRNA template [36]. Thus, the reduction in sequence length of highly expressed genes is more likely to be compatible with the hypothesis that higher efficiency is required for the expression of highly expressed genes.

In brief, the above results suggest that the shortness of highly expressed genes could hardly be interpreted by the energy-cost hypothesis, but could be better reconciled with the time-cost hypotheis. More importantly, it is indicated that the reduction in sequence length would not always cause a reduction in energy or time costs for gene expression, because sequence contraction is tightly associated with elevated gene expression. Whether energy/time costs and sequence length consistently decrease with expression level dependents on the way sequence length is related with expression level.

Of course, the assumption that energy cost of gene expression is proportional to the product of sequence length and expression level is somewhat imprecise. More precise state may be that energy cost of the expression of a gene is proportional to the product of sequence length and the total number of synthesized mRNA molecules and/or protein molecules. Apparently, for two genes with same sequence length and same level of expression, the one with longer-lived mRNA/protein molecules would cost less energy. The decay rates of mRNA/protein molecules must therefore be incorporated into the functional relationship between energetic cost, sequence lengths and mRNA/protein abundance. However, most MPSS and microarray experiments of the current stage can not distinguish transcripts of steady-state from those increasingly accumulated or those gradually decayed. As a result, we could not determine whether the detected gene expression levels represent the steady-state abundance of mRNA molecules, or they only probe instantaneous states. Besides, the protein expression levels could only be limitedly predicted by mRNA expression levels [37-39], meaning that it is in fact improper to evaluate energy and time costs for translation based on mRNA expression data. In this way, expression data of finer resolution combined with genome-wide estimations of mRNA/protein decay rates would be helpful to explore the precise relationships between gene expression and structural characteristics, which could be necessary for the precise evaluation of energy/time costs for genes with different expression levels.

Besides the energy and time cost hypotheses, other explanations exist. For example, short sequences may help to reduce the probability of abortive transcription or erroneous splicing [31].

## Conclusion

Previous studies on the relationships between sequence structure and expression level for plant genes generated conflicted results. Here, it is presented that, different aspects of expression pattern of plant genes correlate with different parts of gene structure in distinct ways. Concretely, highly expressed genes are more compact than lowly expressed genes, which is more pronounced when the effects of expression breadth have been factored out. Conversely, when expression level has been controlled, expression breadth tends to positively correlate with sequence length, especially the length of non-coding regions. These trends seems to be common not only between monocots and dicots, but also between multicellular plants and animals. Furthermore, the extreme values of sequence lengths likely scale as power-laws or logrithmic functions of expression levels which could be better reconciled with the time-cost hypothesis, rather than to be interpreted by the energy-cost hypothesis.

## Data and Methods

Genomic sequences and gene annotations for *Arabidopsis *were obtained from The Arabidopsis Information Resource (TAIR, genomic annotation version 7; [40]) and that of rice were got from Rice Genome Annotation Project (version 5.0; [41]). For both plants, transposon genes and psuedogenes were removed from the dataset. When facing the case of alternative splicing, the longest transript was selected. For any gene, if either end (5' or 3') of the transcript overlap with another transcript on the genome, the corresponding intergenic region was set to 'NA'.

The Massively Parallel Signature Sequencing (MPSS) expression data for *Arabidopsis *and rice were downloaded from plant MPSS databases [42]. These data contain estimates of the number of short unique sequence tags from messenger RNAs [19]. Expression information came from 17 *Arabidopsis *tissues and 18 rice tissues (for library information, see Additional File 7). The downloaded datasets contain mappings of short tags to *Arabidopsis *locus identifier, which were subsequently used to obtain expression estimates for genes. For each rice gene, short tags were compared with the sequence of transcript units annotated in the TIGR5.0 database, and tags exactly mapped to some part of the transcript in the sense manner were taken as representatives of that gene. To obtain expression estimate for each gene, the abundance of tags representing the corresponding gene were averaged, with tags simultaneously mapped to multiple genes discarded. The cutoff for the determination of expression of a gene in a given tissue was set to 5 transcripts per million, to avoid false positive detection of expression. Eventually, MPSS expression data were alailable for 23,535 protein-coding genes in *Arabidopsis *and 26,016 protein-coding genes in rice.

Microarray data for *Arabidopsis *genes were from the experiments published by Schmid et al. [20], which provided a good summarization of global expression profile for 79 *Arabidopsis thaliana *tissues (for detailed information, see Additional File 8). This dataset was downloaded from the website of TAIR. Replicated expression data of different probes and arrays corresponding to the same gene were averaged, using probe to gene mapping relationships supplied by TAIR (TAIR7). Expression values of probes corresponding to multiple genes simultaneously were discarded. The median expression value for all genes across all tissues was adopted as the cutoff to determine whether a gene is expressed in a tissue. Eventually, the dataset contains expression information for 20,460 protein-coding genes.

Rice microarray data were downloaded from the Gene Expression Omnibus (GEO) database of NCBI [43], by the platform ID GPL2025. These oligonucleotide array experiments were performed based on the platform Affymetrix GeneChip Rice Genome Array (for platform information, see [44]). Only expression data from experiments performed using wild-type tissues under normal biochemical conditions were used. In total, 35 different normal samples were available for rice genes (for sample information, see Additional File 9). Following the same procedure as in *Arabidopsis*, gene-level expression values were generated. At last, expression information were available for 25,482 protein-coding genes.

The statistical analyses and all plotting were implemented using the language and environment software R [45].

## Competing interests

The author declares that they have no competing interests.

## Reviewers' comments

### Reviewer's report 1

I. King Jordan, School of Biology, Georgia Institute of Technology, Atlanta, Georgia 30306, USA.

In this paper, Hongxing Yang presents an analysis of the relationship between gene expression and various gene structural features for the plants *Arabidopsis thaliana *and *Oryza sativa *(rice). I support the publication of this manuscript in Biology Direct as it deals with an active area of investigation regarding gene regulation and addresses some unresolved questions in functional genomics. Furthermore, while the analyses are relatively straightforward, the work does appear to be technically sound. Indeed, one of the strengths of the approach used here is the use of two distinct sources, sequence based and microarray based, of high-throughput gene expression data. The consistency of the results using both sources of data underscores their reliability.

In essence, Yang has correlated gene expression parameters, overall and average expression levels along with expression breadth, with a number of gene structural features, such as gene length, CDS length, intron length and number. He finds that highly expressed genes are more compact, while broadly expressed genes have longer non-coding sequences. These results stand in contrast to previous work that showed highly expressed Arabidopsis and rice genes were less compact [14]. The implications of the results reported here are discussed at some length and specifically considered with respect to two models explaining the relationship between gene expression and structure: the energy-cost versus time-cost hypotheses.

One concern with the paper is that readers may be left with impression that there is an imbalance between the amount of results reported and their discussion. The results are fairly succinct and they are interpreted at some length. Furthermore, these results consist solely of correlations between features and the interpretations of the correlations are pushed to the limit in terms of what they may be able to explain biologically. To be fair to the author, it should be pointed out that a substantial amount of work and analysis was done, much of which can be found in the supplement. However, the substance of these analyses is fairly narrow. The manuscript would benefit from some caution in terms of interpretation of results and articulation of possible alternative explanations. If specific testable predictions regarding the authors interpretation could be made, that would help as well.

The magnitudes of the correlation coefficients reported here, while statistically significant, are rather low. The spread in the data is even further evident in Figure 1. These kinds of patterns are not unusual for genome scale comparisons of the kind reported here. However, the author's interpretation that the low correlation values are due to non-linear relationships between gene expression and length does not yield any biological insight. It simply quite likely that gene expression and gene structure are influenced by numerous factors, only a few of which have been interrogated here.

The use of the average expression levels along with partial correlation coefficients are used to try and tease apart effects based on the level of expression from those due to the breadth of expression. This analysis is OK, but a more rigorous multiple regression or perhaps a principle components analysis could be used to reveal more about the nature of the signal observed here and the strongest relationships between gene expression and structural features.

In the introduction, the study of Ren et al. [14] is cited as demonstrating that highly expressed genes are longer than lowly expressed ones in Arabidopsis and rice. However, on page 5, the same study is cited as demonstrating that "highly expressed genes in flowering plants are more compact [14]." I think the latter sentence represents a misstatement that should be corrected. More to the point, the difference between the data obtained in this study and the results of Ren et al. are attributed to the analytical approach taken in the previous report, specifically the binning procedure. In order to support this assertion, it would be desirable to perform the analysis using the approach described by Yang here on the data used by Ren et al. The results should change to be consistent with what is seen in this paper if the difference is due to the distinct analytical approaches in the two works. I believe that it should be worthwhile to do such a re-analysis of the Ren et al. data since the differences between that earlier study and this work are central to the message of this paper.

Author's response

I would like to thank Dr. Jordan for his comments on this manuscript. Following is my response. Firstly, I really appreciate Dr. Jordan's suggestions that alternative explanations of the results should be explored. There may exist some other mechanisms, possibly behind the biochemical activities of transforming DNA molecules to mRNA and subsequently to protein molecules, that determine the relationship between gene structure and expression. One previous research has made an effort toward this direction. Alternative explanations put forwar include, for example, highly expressed genes need to maintain compact structure to reduce the chances of premature termination of the elongation process during transcription, or to save space in the interchromatin compartment [31]. Secondly, I conducted principal components analysis to investigate the relationship between gene structure and expression in a more rigorous way. The results were presented in Additional File 2. Apparently, this analysis gives the same scenario as the partial correlation analysis. Thirdly, I re-analyzed the microarray data for *Arabidopsis *genes used by Ren et al. with the analyzing strategies adopted in the current study. Shown in Table S5 (see Additional File 10), this analysis confirmed the findings of the current study.

### Reviewer's report 2

Liran Carmel, Department of Genetics, The Alexander Silberman Institute of Life Sciences, Faculty of Science, The Hebrew University of Jerusalem, Edmond J. Safra Campus, Givat Ram, Jerusalem 91904, Israel (nominated by Dr. Eugene V. Koonin).

The manuscript addresses an interesting question. Its proper understanding may shed light on key factors designing gene structure in multicellular organisms. I think the manuscript reports some important findings, although perhaps not always emphasized enough.

Author's response

I thank Dr. Koonin for his nomination of a reviewer and Dr. Liran Carmel for his comments. Below I response to the questions respectively.

1. The author finds that when controlling expression level, broadly expressed genes are less compact, at least in the noncoding part. This is more of less exactly the opposite than would have been expected from the genomic design hypothesis. The author reports this finding a number of times along the manuscript and mentions some of its consequences but, in my eyes, he does not state out clearly that this is a strong refutation of the genomic design hypothesis.

Author's response

The correlations between gene structure and expression level indeed strongly conflict with the hypothesis of genomic design. However, the trend that more broadly expressed genes tend to contain longer non-coding sequences support the view that gene function and expression broadness significantly influence each other. In principle, this view is consistent with the genomic design hypothesis, though the underlying biological mechanisms may not follow this hypothesis.

2. I feel somewhat inconvenient that the author includes intron number as one of the measures to gene compactness. While intron length (whether average or total) was demonstrated a number of times to be negatively correlated with expression level and breadth, the behavior of intron number as well as its impact on the compactness is less clear. First, since the length of the coding part varies considerably between genes, it is more meaningful to talk about intron density rather than intron number. Second, evidence is accumulating that intron density increases, at least for human and Arabidopsis, with expression level and breadth (Fahey and Higgins 2007, J. Mol. Evol. 65:349-357; Carmel et al. 2007, Genome Res. 17:1045-1050). Third, the relevance of intron number to either the selection for efficiency or genomic design is of minor importance. Of much greater relevance is the total length of the introns.

Author's response

Intron density is of course a very important factor. However, intron number in fact could be viewed as a representative of the energy/time cost of splicing processes, and thus should be directly examined to test the selection for efficiency model. Moreover, as what has been unraveled by this study (see also [21]), the level and broadness of gene expression have distinct influences on different parts of gene regions. In this respect, intron density may entangle the analysis of the subjects considered here. Nevertheless, when it was examined using the analyzing strategies of this study, intron density was found to negatively correlate with *Exp*_*avg *_and positively with *Width*, consistent with the pattern of other parameters (see Table 1 and Additional file 1).

3. The author mentions that "intron intensity" is positively correlated with expression level for *Ostreococcus lucimarinus*. It is not clear to me what does it mean.

Author's response

This is a typo and has been corrected.

4. *Exp*_*tot *_should, almost by definition, be highly correlated with expression breadth. The author devotes an entire paragraph to show that, and eventually concludes that *Exp*_*avg *_is better as a proxy for expression level. I think that this point is clear to begin with, and that *Exp*_*tot *_should not be mentioned in the manuscript at all.

Author's response

The reason for dealing with *Exp*_*tot *_seriously is that, previous researchers haven't clearly distinguished *Exp*_*tot *_and *Exp*_*avg*_, especially when the correlations with gene structure were investigated.

5. At least for MPSS experiments in Arabidopsis, the author used samples of treated plants (see Table S2). I think that for the purpose of the current manuscript, only untreated samples should be taken.

Author's response

After removing the data from treated plants, the results remained essentially the same.

6. Methodologically, I am a bit bothered by the underlying assumption of the author that expression levels of a gene in different tissues are comparable. This is not necessarily the case, and this is an important reason why Ren et al. ranked the genes in each sample prior to averaging along the samples.

Author's response

At least for microarray data, previous studies [46-48] have shown that good consistency exists between gene expression measurements produced within the same laboratory using commercial platforms (such as the microarray data used by this study [20]). Moreover, I have also computed the correlations between gene structure and ranked expression levels, shown in Table S6 (see Additional File 11). It can be seen that, gene expression ranks and gene expression levels show the same trend with respect to their correlations with gene structure. At least for the issues discussed here, the comparability of gene expression measurement between different experiments doesn't seem to be a problem.

7. The author computes an approximate dependence of the maximum energy cost as a function of the expression level, and uses it to favor the time-cost hypothesis over the energy-cost one. Regardless of the fact that the time-cost hypothesis might indeed be more convincing, I am not sure that the model built by the author proves anything. At least, when nothing on the strength of the selective pressures of time versus energy is known.

Author's response

The fact presented here could not tell us with a definite manner which model, energy-cost or time-cost, plays the major role in the adapted evolution of gene structure and expression. The main aim is that, it presents a piece of evidence which would help to test the hypotheses made by previous researchers. What should be noted is, shorter genes consume fewer energy is only an intuitive judgment, which overlooks the fact that evolution of gene structure and expression pattern are tightly entangled together. Nevertheless, one model should receive greater support if its predictions conform to the data better than the other. As for the selective strengths, it has been shown that selective pressures for saving energy may be very minor [31]. Hope future studies could find a solution to directly compare the selective pressures regard to time versus energy cost.

8. The author determines whether a gene is present in a tissue or not based on a cutoff that is the median expression value for all genes across all tissues. Isn't there a way to compute a different cutoff per sample?

Author's response

Using a common cutoff for all tissues is the most direct and acceptable way to compare gene expressions across tissues, especially when we don't have exact measures about the total quantity of expressed transcripts per tissue.

### Reviewer's report 3

Fyodor A. Kondrashov, Bioinformatics and Genomics Programme, Centre for Genomic Regulation, 08003 Barcelona, Spain.

Since the publication of the hypothesis that nonfunctional intron sequence may be under selection for increased transcriptional efficiency this issue has gathered considerable momentum. An increasing fraction of "junk DNA" is associated with a function and as a result the debate of selection in intron sequences transitioned to a standoff between opposing "efficiency" and "design" models. At the same time the efficiency model has been increasingly associated with the economy of energy in the course of transcription. In addition to the experimental data of the relationship between intron length and level of expression in plants the present manuscript presents an excellent overview of the conceptual side of the issue of selection in introns. Firstly, the author is absolutely right that the efficiency and design models are not mutually exclusive. The concept that some large fraction of some introns may be under selective constraint for a specific function does not negate the possibility that neutral segments of the same intron may be under selection for transcriptional efficiency. Indeed, intron sequence in *S. serevisiae *are likely to be under selection for function, however, negative selection against increase of their length is likely to be strong. Second, within the efficiency model the author correctly distinguishes selection for energy and time conservation. While the modern literature increasingly associates the efficiency model with energetic constraint I am of the opinion that constraints on time of large introns will prove to be much more substantial. For these reasons I am delighted to see this balanced paper presented in Biology Direct and hope that, in the future, scientists investigating selection in intronic DNA would take a leaf out of Dr. Yang's book and conduct their studies consistently with the two points raised above.

Author's response

I should express my sincere appreciation for Dr. Kondrashov's encouraging comments and hope this study could contribute to the discussion of the co-adapted evolution of gene structure and expression.

## Supplementary Material

Additional file 1 **Table S1.pdf**. Spearman's rank sum correlations between expression pattern (microarray data) and structural parameters for *Arabidopsis *and rice genes. For each structural parameter, the first line represents the corrleations with expression pattern, while the second line represents partial correlations. Controlled variable for the columns of *Exp*_*avg *_is expression breadth and that for the columns of *Width *is average expression level. *Exp*_*tot*_, total expression level; *Exp*_*avg*_, average expression level; *Width*, expression breadth; CDS, Coding Sequence; UTR, Untranslated Region. ***, *P *< 1*e *- 10;**, 1*e *- 10 <*P *< 1*e *2; *, 0.01 <*P *< 0.05. Click here for file

Additional file 2 **Fig S1.pdf**. Principal components analysis of the correlation between sequence structural parameters and gene expression. Points represent genes, while arrows represnt variables. In each graph, if the angle between two arrows is > 90°, the two variables represented by these arrows are negatively correlated, while if the angle is < 90°, the variables are positively correlated. These figures were produced using expression data from MPSS experiments. Using data from microarray data gives similar pictures. Click here for file

Additional file 3 **Fig S2.pdf**. Boxplots of structural characteristics versus expression level (microarray data) for *Arabidopsis *and rice genes. Boxes represent the range of parameters for each gene group, with bold central lines represent the medians, lower and upper boundaries represent the first and third quartiles respectively, whereas whiskers extend to the most extrem points within 1.5× interquartile ranges from the boxes. The red curves represent mean values of parameters for each gene group, whereas horizontal darkviolet lines indicate the population median for each structural parameter. Presented parameters are: CDS length in (a) *Arabidopsis *and (b) rice; total intron length per gene in (c) *Arabidopsis *and (d) rice; number of introns per gene in (e) *Arabidopsis *and (f) rice. Differences in structural parameters between different expression groups are statistically significant (all Kruskal-Wallis rank sum test *P *< 2e-16). Click here for file

Additional file 4 **Fig S3.pdf**. Boxplots of structural characteristics versus expression breadth for *Arabidopsis *and rice genes. Boxes represent the range of parameters for each gene group, with bold central lines represent the medians, lower and upper boundaries represent the first and third quartiles respectively, whereas whiskers extend to the most extreme points within 1.5× interquartile ranges from boxes. The red curves represent mean values of parameters for each gene group, whereas horizontal dotted lines indicate the median of the population for each parameter. Presented parameters are: number of introns per gene in (a) *Arabidopsis *and (b) rice; total intron length per gene in (c) *Arabidopsis *and (d) rice; length of CDS in (e) *Arabidopsis *and (f) rice. Differences in structural parameters between different expression groups are statistically significant (all Kruskal-Wallis rank sum test *P *< 2e-16). Click here for file

Additional file 5 **Fig S4.pdf**. extreme transcript lengths versus expression levels (microarray data) for plant genes. Figure (a), extreme transcript lengths of *Arabidopsis *genes scale as a power-law of average expression level; Figure (b)-(f), extreme transcript lengths of *Arabidopsis *and rice genes scale as logrithmic functions of expression levels. In each figure, points represent the whole dataset, whereas triangles represent data subset used to fit the dark-violet linear line; dashed red curve represents the extreme energy-cost of transcription; dotted vertical line indicates the maximum point of the energy-cost curve. Equations show the functional form for corresponding curves. Figures at the left side represent *Arabidopsis *genes, whereas that at the right side represent rice genes. The adjusted r-squares for the linear regression analyses range from 0.80 to 0.91, and analyses of variance indicate high statistical significance (all *P*-value < 2e-16). Similar trends could be observed for other structural parameters, such as total intron length per gene and intron number per gene. Click here for file

Additional file 6 **Fig S5.pdf**. Extreme energy-/time- costs for the expression of plant genes vary with expression level (microarray data). Under the assumption that extreme sequence lengths scale as logrithmic functions of expression level, the black solid curve shows how the extreme energy-cost will change with expression level, while other curves indicate the trends of time-cost, which is assumed to scale as sublinear functions (with *α *being the scaling factor) of expression level. It is shown that, smaller *α *implies higher effciency requirements for highly expressed genes. Y-axis represents the scale of energy-cost, while the numerical values of time-cost have been scaled to the same range for the convenience of comparison. a = 66094, b = 3494, taken from the case of extreme transript lengths versus total expression level for *Arabidopsis *genes. Scenarios for other cases are essentially the same. Click here for file

Additional file 7 **Table S2.pdf**. Library information for MPSS expression data. Click here for file

Additional file 8 **Table S3.pdf**. Sample information for *Arabidopsis *microarray data. Click here for file

Additional file 9 **Table S4.pdf**. Sample information for rice microarray data. Click here for file

Additional file 10 **Table S5.pdf**. Correlation between expression pattern and sequence structural parameters for *Arabidopsis *genes. The expression data are the microarray data Ren et al. (2006) used in their study. For each structural parameter, *ρ*s represent Spearman's rank sum corrleation coefficients between expression pattern and structural parameters, while partial *ρ*s represent Spearman's partial correlations. Controlled variable for *Exp*_*avg *_is expression width and that for *Width *is average expression level. *Exp*_*tot*_, total expression level; *Exp*_*avg*_, average expression level; *Width*, expression breadth. CDS, Coding Sequence; UTR, Untranslated Region. Level of significance: *, *P *> 0.05; **, 0.001 <*P *< 0.05; ***, 1*e *- 10 <*P *< 1*e *- 3; No asterisks indicates *P *< 1*e *- 10. Numbers in bold indicate highly significant partial correlations (*P *< 1*e *- 10). Click here for file

Additional file 11 **Table S6.pdf**. The correlations between expression pattern and sequence structural parameters for *Arabidopsis *and rice genes. Genes were separately sorted according to their expression levels in each library; the ranks for each gene were then averaged to give the value of *Exp*_*avg*_. Notably, in each library, a gene was taken as expressed only when > = 5 tags could be mapped onto it. For each structural parameter, the first line shows Spearman's rank sum corrleations with expression pattern, while the second line shows Spearman's partial correlations. Controlled variable for the columns of *Exp*_*avg *_is expression width and that for the columns of *Width *is average expression level. CDS, Coding Sequence; UTR, Untranslated Region. Level of significance: *, *P *> 0.05; **, 0.001 <*P *< 0.05; ***, 1*e *- 10 <*P *< 1*e *- 3; No asterisks indicates *P *< 1*e *- 10. Numbers in bold indicate highly significant partial correlations (*P *< 1*e *- 10). Click here for file
