# Supplementary material for: In plants, expression breadth and expression level distinctly and non-linearly correlate with gene structure
Source: Biol Direct. 2009 Nov 21;4:45. doi: 10.1186/1745-6150-4-45 (PMC2794262; doi:10.1186/1745-6150-4-45)
Supplement: Additional file 1 — Table S1.pdf. Spearman's rank sum correlations between expression pattern (microarray data) and structural parameters for Arabidopsis and rice genes. For each structural parameter, the first line represents the corrleations with expression pattern, while the second line represents partial correlations. Controlled variable for the columns of Expavg is expression breadth and that for the columns of Width is average expression level. Exptot, total expression level; Expavg, average expression level; Width, expression breadth; CDS, Coding Sequence; UTR, Untranslated Region. ***, P < 1e - 10;**, 1e - 10 <P < 1e 2; *, 0.01 <P < 0.05. [file 1745-6150-4-45-S1.PDF]

Table S1 - The correlations between expression pattern and sequence structural parameters for *Arabidopsis* and rice genes.

Parameters	Arabidopsis			Rice		
	Exp_{tot}	Exp_{avg}	$Width$	Exp_{tot}	Exp_{avg}	$Width$
Length of primary transcript	0.187	-0.091	0.288	0.139	0.134	0.234
		**-0.228**	**0.286**		**-0.100**	**0.216**
Length of CDS	-0.027	-0.184	0.048	-0.064	-0.071	0.012*
		**-0.203**	**0.090**		**-0.136**	**0.116**
Average exon length	-0.142	-0.065	-0.184	-0.188	-0.167	-0.220
		0.038***	**-0.215**		-0.0005*	**-0.157**
Average intron length	0.205	0.071	0.251	0.161	0.165	0.222
		**-0.054**	**0.225**		-0.033***	**0.157**
Number of introns	0.221	-0.002*	0.303	0.272	0.260	0.343
		**-0.151**	**0.282**		-0.032***	**0.228**
Intron density	0.266	0.102	0.321	0.317	0.307	0.360
		**-0.058**	**0.286**		0.027***	**0.194**
Total intron length	0.243	0.016*	0.333	0.259	0.254	0.343
		**-0.158**	**0.320**		**-0.046**	**0.240**
5' UTR length	0.472	0.146	0.507	0.112	0.113	0.184
		**-0.052**	**0.331**		**-0.048**	**0.153**
3' UTR length	0.473	0.145	0.509	0.217	0.199	0.260
		**-0.055**	**0.336**		-0.003*	**0.165**
5' intergenic length	-0.078	-0.022**	-0.124	0.009*	-0.011*	-0.013**
		**0.066**	**-0.167**		0.038***	-0.039***
3' intergenic length	-0.129	-0.011*	-0.167	-0.099	-0.089	-0.120
		**0.062**	**-0.142**		0.012*	**-0.078**

For each structural parameter, the first line shows Spearman's rank sum corrleation coefficients with expression pattern, while the second line shows Spearman's partial correlations. Controlled variable for the columns of Exp_{avg} is expression width and that for the columns of $Width$ is average expression level. Intron density was defined as the ratio of intron number to coding sequence length, i.e. the number of introns per coding base for each gene. Exp_{tot}, total expression level; Exp_{avg}, average expression level; $Width$, expression breadth. CDS, Coding Sequence; UTR, Untranslated Region. Level of significance: *, $P > 0.05$; **, $0.001 < P < 0.05$; ***, $1e-10 < P < 1e-3$; No asterisks indicates $P < 1e-10$. Numbers in bold indicate highly significant partial correlations ($P < 1e-10$).
