# Supplementary material for: In plants, expression breadth and expression level distinctly and non-linearly correlate with gene structure
Source: Biol Direct. 2009 Nov 21;4:45. doi: 10.1186/1745-6150-4-45 (PMC2794262; doi:10.1186/1745-6150-4-45)

Fig. S1 - Principal components analysis of the correlation between sequence structural parameters and gene expression.

(a), *Arabidopsis*; (b), rice. In each graph, points represent genes, while arrows represnt variables. If the angle between two arrows is $> 90°$, the two variables represented by these arrows are negatively correlated, whereas if the angle is $< 90°$, the variables are positively correlated. These figures were produced using expression data from MPSS experiments. Using data from microarray data gives similar pictures.

(a) *Arabidopsis*

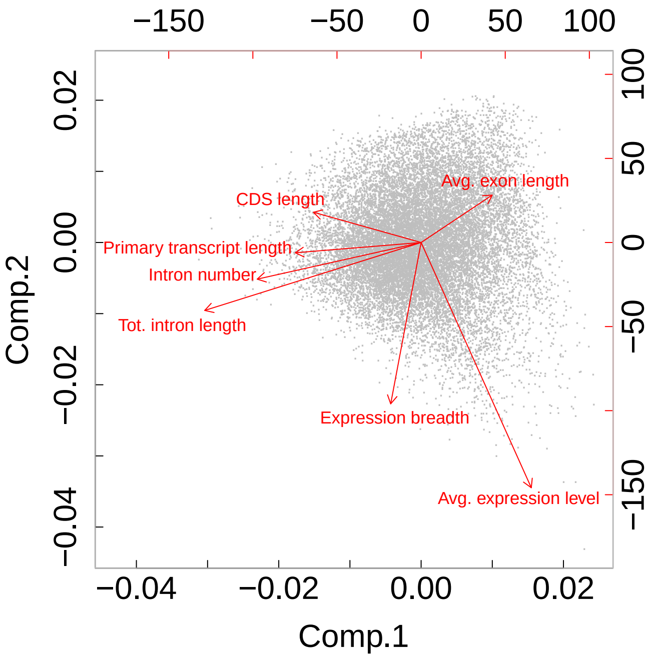

(b) Rice

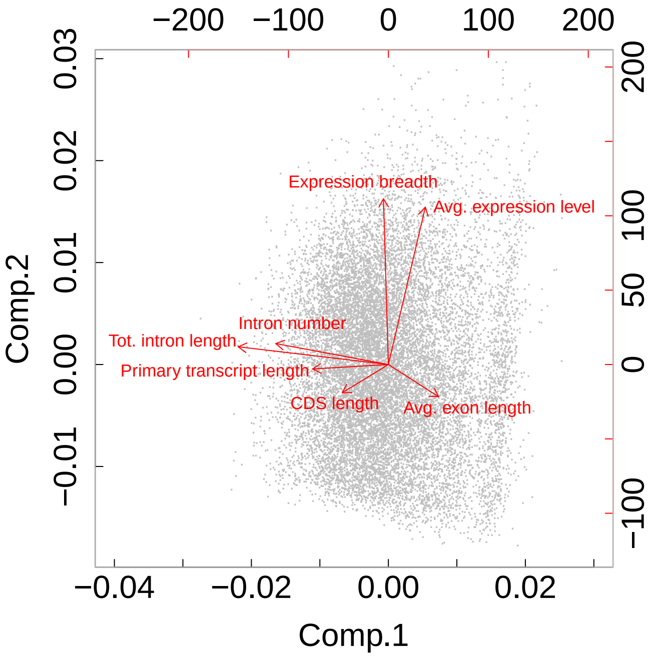

Supplement: Additional file 2 — Fig S1.pdf. Principal components analysis of the correlation between sequence structural parameters and gene expression. Points represent genes, while arrows represnt variables. In each graph, if the angle between two arrows is > 90°, the two variables represented by these arrows are negatively correlated, while if the angle is < 90°, the variables are positively correlated. These figures were produced using expression data from MPSS experiments. Using data from microarray data gives similar pictures. [file 1745-6150-4-45-S2.PDF]
