# Supplementary material for: In plants, expression breadth and expression level distinctly and non-linearly correlate with gene structure
Source: Biol Direct. 2009 Nov 21;4:45. doi: 10.1186/1745-6150-4-45 (PMC2794262; doi:10.1186/1745-6150-4-45)
Supplement: Additional file 3 — Fig S2.pdf. Boxplots of structural characteristics versus expression level (microarray data) for Arabidopsis and rice genes. Boxes represent the range of parameters for each gene group, with bold central lines represent the medians, lower and upper boundaries represent the first and third quartiles respectively, whereas whiskers extend to the most extrem points within 1.5× interquartile ranges from the boxes. The red curves represent mean values of parameters for each gene group, whereas horizontal darkviolet lines indicate the population median for each structural parameter. Presented parameters are: CDS length in (a) Arabidopsis and (b) rice; total intron length per gene in (c) Arabidopsis and (d) rice; number of introns per gene in (e) Arabidopsis and (f) rice. Differences in structural parameters between different expression groups are statistically significant (all Kruskal-Wallis rank sum test P < 2e-16). [file 1745-6150-4-45-S3.PDF]

Fig. S2 - Boxplots of structural characters versus expression level (microarray data) for *Arabidopsis* and rice genes.

In each graph, x-axis represents gene-expression level, boxes represent the value range of parameters for each gene group, with bold central lines represent the medians, lower and upper boundaries represent the first and third quartiles respectively, whereas whiskers extend to the most extrem points within 1.5× interquartile range from the boxes. Horizontal darkviolet lines indicate the population median for each parameter. Presented parameters are: CDS length in (a) *Arabidopsis* and (b) rice; total intron length per gene in (c) *Arabidopsis* and (d) rice; number of introns per gene in (e) *Arabidopsis* and (f) rice. Differences in structural parameters between different expression groups are statistically significant (all Kruskal-Wallis rank sum test P <2e-16).

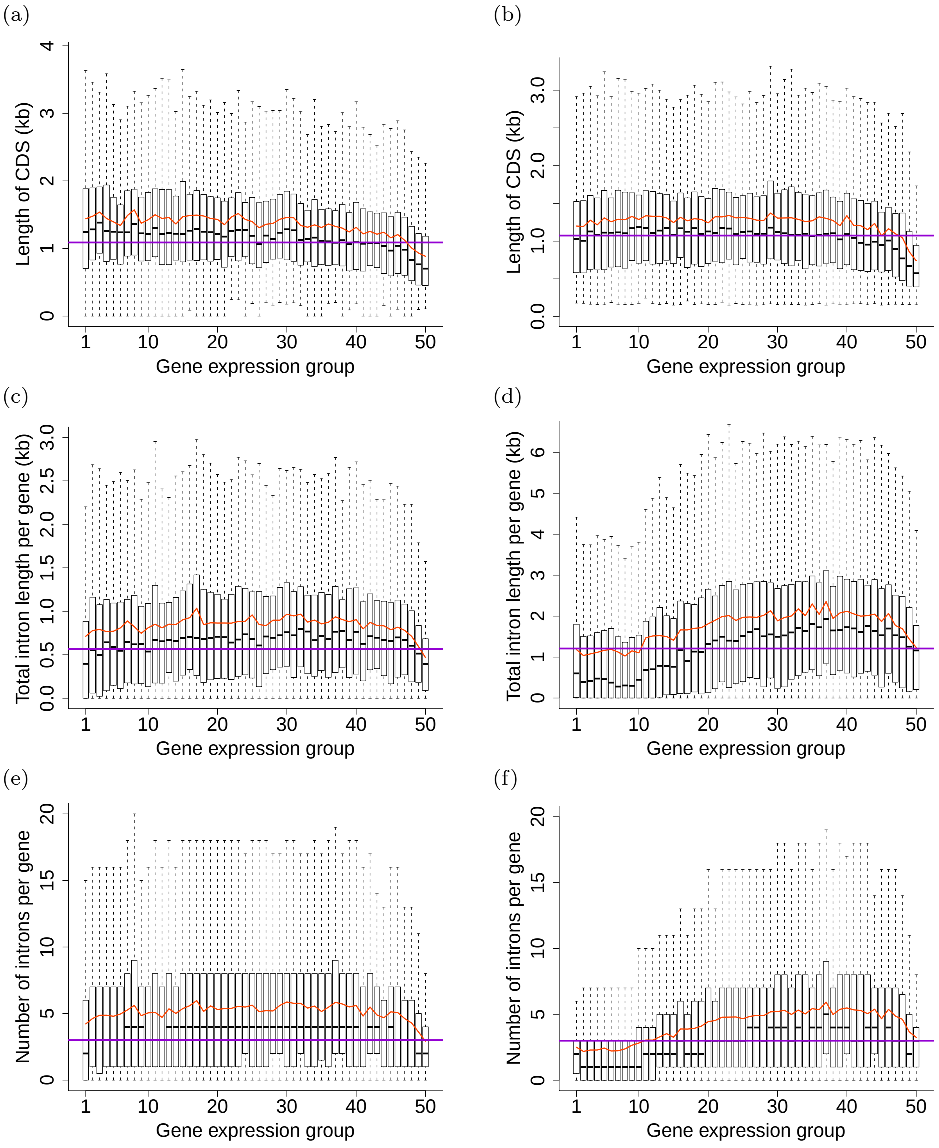
(a)
Length of CDS (kb)
Gene expression group
(b)
Length of CDS (kb)
Gene expression group
(c)
Total intron length per gene (kb)
Gene expression group
(d)
Total intron length per gene (kb)
Gene expression group
(e)
Number of introns per gene
Gene expression group
(f)
Number of introns per gene
Gene expression group
