# Supplementary material for: In plants, expression breadth and expression level distinctly and non-linearly correlate with gene structure
Source: Biol Direct. 2009 Nov 21;4:45. doi: 10.1186/1745-6150-4-45 (PMC2794262; doi:10.1186/1745-6150-4-45)
Supplement: Additional file 4 — Fig S3.pdf. Boxplots of structural characteristics versus expression breadth for Arabidopsis and rice genes. Boxes represent the range of parameters for each gene group, with bold central lines represent the medians, lower and upper boundaries represent the first and third quartiles respectively, whereas whiskers extend to the most extreme points within 1.5× interquartile ranges from boxes. The red curves represent mean values of parameters for each gene group, whereas horizontal dotted lines indicate the median of the population for each parameter. Presented parameters are: number of introns per gene in (a) Arabidopsis and (b) rice; total intron length per gene in (c) Arabidopsis and (d) rice; length of CDS in (e) Arabidopsis and (f) rice. Differences in structural parameters between different expression groups are statistically significant (all Kruskal-Wallis rank sum test P < 2e-16). [file 1745-6150-4-45-S4.PDF]

Fig. S3 - Boxplots of structural parameters versus expression breadth for *Arabidopsis* and rice genes.

In each graph, boxes represent the range of the structural values for each expression group, with bold central lines represent the medians, lower and upper boundaries represent the first and third quartiles respectively, whereas whiskers extend to the most extrem points within $1.5\times$ interquartile range from the boxes. Horizontal dotted lines indicate the population median for each parameter. Presented parameters are: number of introns per gene in (a) *Arabidopsis* and (b) rice; total intron length per gene in (c) *Arabidopsis* and (d) rice; CDS length in (e) *Arabidopsis* and (f). Differences in structural parameters between different expression groups are statistically significant (all Kruskal-Wallis rank sum test $P <$2e-16).

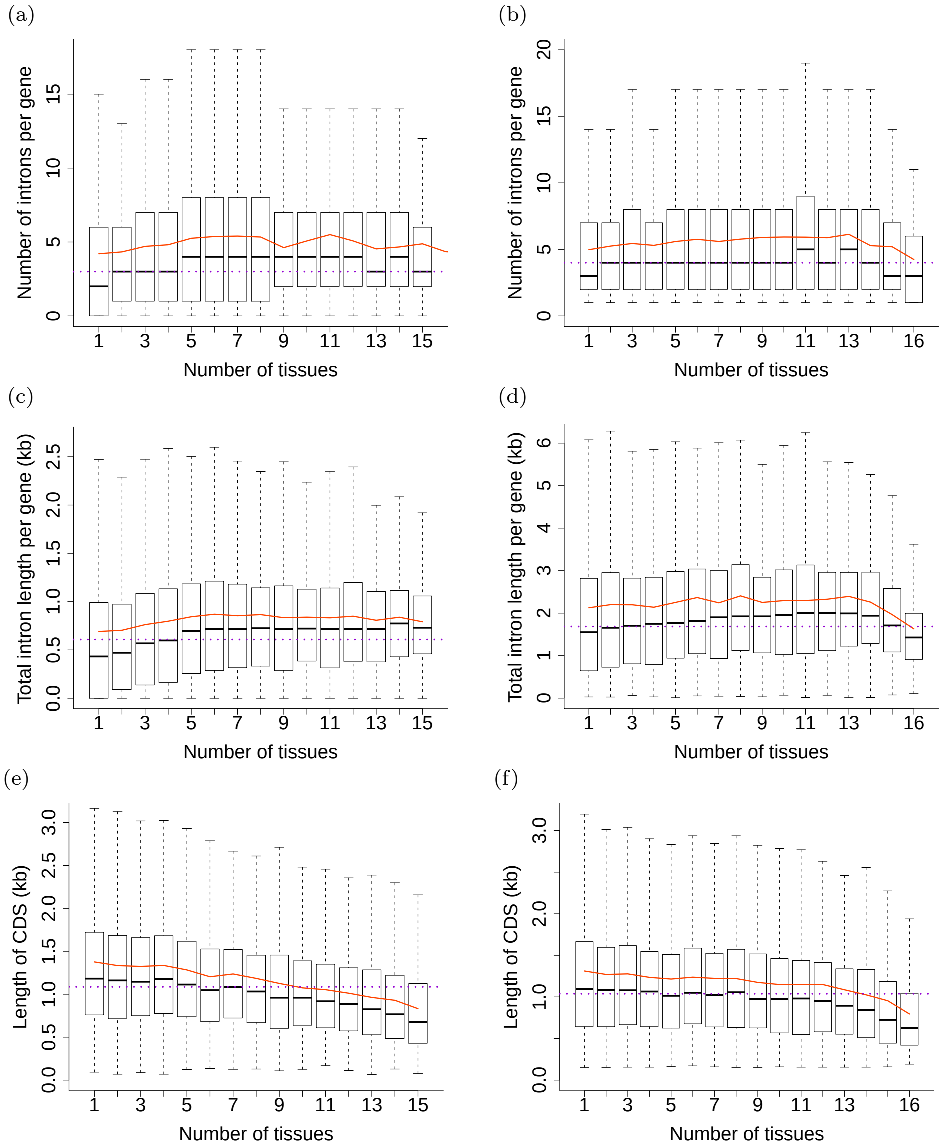
(a)
Number of introns per gene
Number of tissues
(b)
Number of introns per gene
Number of tissues
(c)
Total intron length per gene (kb)
Number of tissues
(d)
Total intron length per gene (kb)
Number of tissues
(e)
Length of CDS (kb)
Number of tissues
(f)
Length of CDS (kb)
Number of tissues
