# Supplementary material for: In plants, expression breadth and expression level distinctly and non-linearly correlate with gene structure
Source: Biol Direct. 2009 Nov 21;4:45. doi: 10.1186/1745-6150-4-45 (PMC2794262; doi:10.1186/1745-6150-4-45)
Supplement: Additional file 5 — Fig S4.pdf. extreme transcript lengths versus expression levels (microarray data) for plant genes. Figure (a), extreme transcript lengths of Arabidopsis genes scale as a power-law of average expression level; Figure (b)-(f), extreme transcript lengths of Arabidopsis and rice genes scale as logrithmic functions of expression levels. In each figure, points represent the whole dataset, whereas triangles represent data subset used to fit the dark-violet linear line; dashed red curve represents the extreme energy-cost of transcription; dotted vertical line indicates the maximum point of the energy-cost curve. Equations show the functional form for corresponding curves. Figures at the left side represent Arabidopsis genes, whereas that at the right side represent rice genes. The adjusted r-squares for the linear regression analyses range from 0.80 to 0.91, and analyses of variance indicate high statistical significance (all P-value < 2e-16). Similar trends could be observed for other structural parameters, such as total intron length per gene and intron number per gene. [file 1745-6150-4-45-S5.PDF]

Fig. S4 - Extreme transcript lengths versus expression levels (microarray data) for plant genes.

Figure (a), extreme transcript lengths of *Arabidopsis* genes scales as a power-law of average expression level; Figure (b)-(f), extreme transcript lengths of *Arabidopsis* and rice genes scale as logrithmic functions of expression levels. In each figure, points represent the whole dataset, whereas triangles represent data points used to fit the darkviolet linear line; dashed red curve represents the extreme energy-cost of transcription; dotted vertical line indicates the maximum point of the energy-cost curve. Equations show the functional form for corresponding curves. Figures at the left side represent *Arabidopsis* genes, whereas that at the right side represent rice genes. The adjusted r-squares for the linear regression analyses range from 0.80 to 0.91, and analyses of variance indicate high statistical significance (P<2e-16 in all cases). Similar trends could be observed for other structural parameters, such as total intron length per gene and intron number per gene.

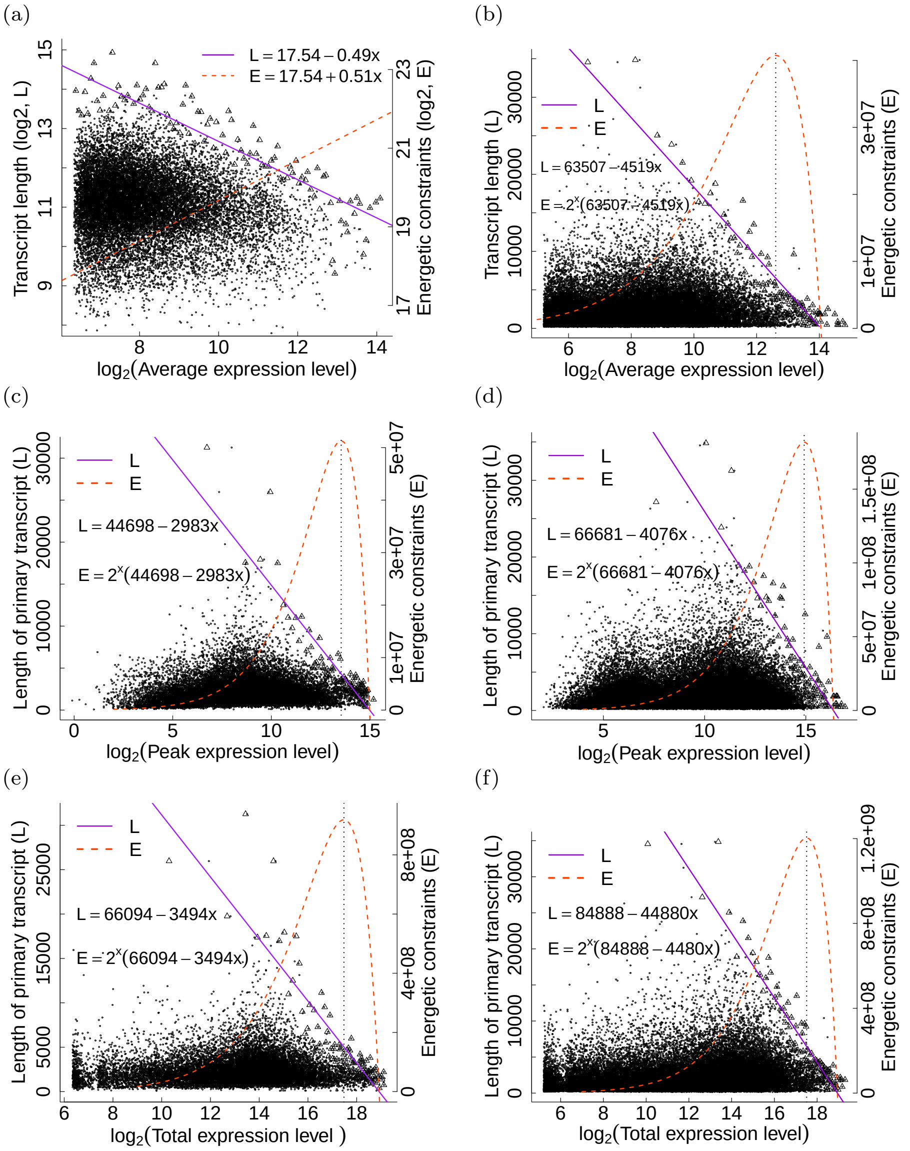
(a)
L = 17.54 – 0.49x
E = 17.54 + 0.51x
Transcript length (log2, L)
Energetic constraints (log2, E)
log2(Average expression level)
(b)
L
E
L = 63507 – 4519x
E = 2x(63507 – 4519x)
Transcript length (L)
Energetic constraints (E)
log2(Average expression level)
(c)
L
E
L = 44698 – 2983x
E = 2x(44698 – 2983x)
Length of primary transcript (L)
Energetic constraints (E)
log2(Peak expression level)
(d)
L
E
L = 66681 – 4076x
E = 2x(66681 – 4076x)
Length of primary transcript (L)
Energetic constraints (E)
log2(Peak expression level)
(e)
L
E
L = 66094 – 3494x
E = 2x(66094 – 3494x)
Length of primary transcript (L)
Energetic constraints (E)
log2(Total expression level)
(f)
L
E
L = 84888 – 44880x
E = 2x(84888 – 4480x)
Length of primary transcript (L)
Energetic constraints (E)
log2(Total expression level)
