# Supplementary material for: In plants, expression breadth and expression level distinctly and non-linearly correlate with gene structure
Source: Biol Direct. 2009 Nov 21;4:45. doi: 10.1186/1745-6150-4-45 (PMC2794262; doi:10.1186/1745-6150-4-45)

Fig. S5 - Extreme values of energy-/time- costs for expression vary with expression level (microarray data) for plant genes.

Under the assumption that extreme sequence lengths scale as logrithmic functions of expression level, the black solid curve shows how the extreme energy-cost will change with expression level, while other curves indicate the trends of time-cost, which is assumed to scale as sublinear functions (with α being the scaling factor) of expression level. It is shown that, smaller α implies higher effciency requirements for highly expressed genes. Y-axis represents the scale of energy-cost, while numerical values of time-cost have been scaled to the same range for the convenience of comparison. a=66094, b= 3494, taken from the case of extreme transript lengths versus total expression level for *Arabidopsis* genes.

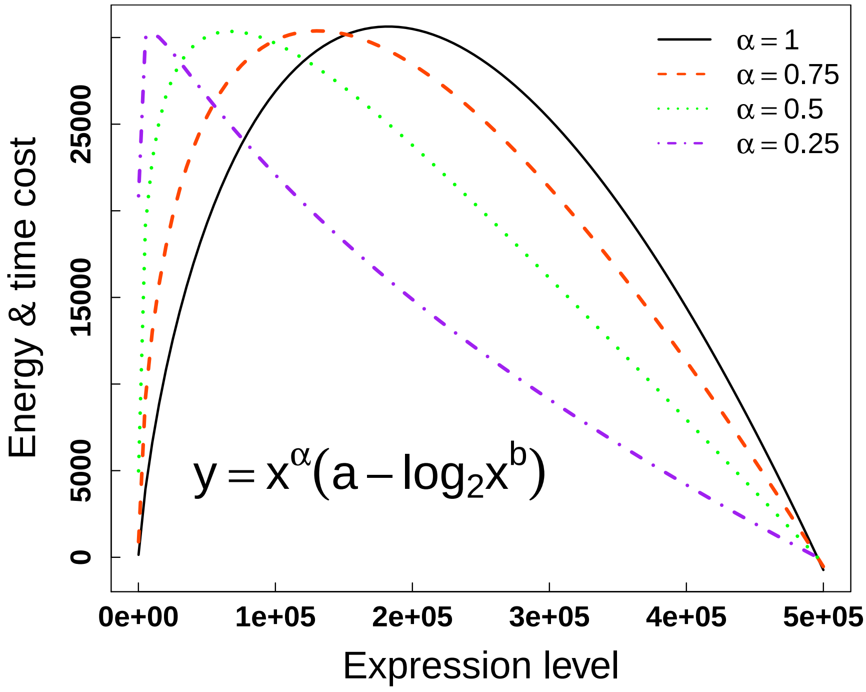

Supplement: Additional file 6 — Fig S5.pdf. Extreme energy-/time- costs for the expression of plant genes vary with expression level (microarray data). Under the assumption that extreme sequence lengths scale as logrithmic functions of expression level, the black solid curve shows how the extreme energy-cost will change with expression level, while other curves indicate the trends of time-cost, which is assumed to scale as sublinear functions (with α being the scaling factor) of expression level. It is shown that, smaller α implies higher effciency requirements for highly expressed genes. Y-axis represents the scale of energy-cost, while the numerical values of time-cost have been scaled to the same range for the convenience of comparison. a = 66094, b = 3494, taken from the case of extreme transript lengths versus total expression level for Arabidopsis genes. Scenarios for other cases are essentially the same. [file 1745-6150-4-45-S6.PDF]
