# Supplementary material for: In plants, expression breadth and expression level distinctly and non-linearly correlate with gene structure
Source: Biol Direct. 2009 Nov 21;4:45. doi: 10.1186/1745-6150-4-45 (PMC2794262; doi:10.1186/1745-6150-4-45)
Supplement: Additional file 7 — Table S2.pdf. Library information for MPSS expression data. [file 1745-6150-4-45-S7.PDF]

Table S2 - Library information for MPSS expression data.

Code	Title
	**Arabidopsis**
CAF	Callus - actively growing, classic MPSS
INF	Infloresence - mixed stage, immature buds, classic MPSS
LEF	Leaves - 21 day, untreated, classic MPSS
ROF	Root - 21 day, untreated, classic MPSS
SIF	Silique - 24 to 48 hr post-fertilization, classic MPSS
AP1	ap1-10 infloresence - mixed stage, immature buds
AP3	ap3-6 infloresence - mixed stage, immature buds
AGM	agamous infloresence - mixed stage, immature buds
INS	Infloresence - mixed stage, immature buds
ROS	Root - 21 day, untreated
SAP	sup/ap1 infloresence - mixed stage, immature buds
LES	Leaves - 21 day, untreated
GSE	Germinating seedlings
CAS	Callus - actively growing, signature MPSS
SIS	Silique - 24 to 48 hr post-fertilization, signature MPSS
	**Rice**
NYR	14 days - Young Roots
NGD	10 days - Germinating seedlings grown in dark
NST	60 days - Stem
NYL	14 days - Young leaves
NME	60 days - Crown vegetative meristematic tissue
NPO	Mature Pollen
NOS	Ovary and mature stigma
NIP	90 days - Immature panicle
NGS	3 days - Germinating seed
NCA	35 days - Callus
NSR	14 days - Young roots stressed in 250 mM NaCl for 24h
NSL	14 days - Young leaves stressed in 250 mM NaCl for 24h
NDR	14 days - Young roots stressed in drought for 5 days
NDL	14 days - Young leaves stressed in drought for 5 days
NCR	14 days - Young roots stressed in 4C cold for 24h
NCL	14 days - Young leaves stressed in 4C cold for 24h
NR2	60 days - Mature Roots - Replicate A & B
NL4	60 days - Mature Leaves - Replicate A, B, C and D

Note: For rice, NR2 and NL4 represent the combination of two and four replicate experiments, respectively.
