# Supplementary material for: In plants, expression breadth and expression level distinctly and non-linearly correlate with gene structure
Source: Biol Direct. 2009 Nov 21;4:45. doi: 10.1186/1745-6150-4-45 (PMC2794262; doi:10.1186/1745-6150-4-45)
Supplement: Additional file 8 — Table S3.pdf. Sample information for Arabidopsis microarray data. [file 1745-6150-4-45-S8.PDF]

Table S3 - Sample information for *Arabidopsis* microarray data[a]

Sample ID	Genotype	Tissue	Age
ATGE_1	Wt	cotyledons	7 days
ATGE_2	Wt	hypocotyl	7 days
ATGE_3	Wt	roots	7 days
ATGE_4	Wt	shoot apex, vegetative + young leaves	7 days
ATGE_5	Wt	leaves 1 + 2	7 days
ATGE_6	Wt	shoot apex, vegetative	7 days
ATGE_7	Wt	seedling, green parts	7 days
ATGE_8	Wt	shoot apex, transition (before bolting)	14 days
ATGE_9	Wt	roots	17 days
ATGE_10	Wt	rosette leaf #4, 1 cm long	10 days
ATGE_11	gl1-T	rosette leaf #4, 1 cm long	10 days
ATGE_12	Wt	rosette leaf # 2	17 days
ATGE_13	Wt	rosette leaf # 4	17 days
ATGE_14	Wt	rosette leaf # 6	17 days
ATGE_15	Wt	rosette leaf # 8	17 days
ATGE_16	Wt	rosette leaf # 10	17 days
ATGE_17	Wt	rosette leaf # 12	17 days
ATGE_18	gl1-T	rosette leaf # 12	17 days
ATGE_19	Wt	leaf 7, petiole	17 days
ATGE_20	Wt	leaf 7, proximal half	17 days
ATGE_21	Wt	leaf 7, distal half	17 days
ATGE_22	Wt	entire rosette after transition to flowering, but before bolting	21 days
ATGE_23	Wt	entire rosette after transition to flowering, but before bolting	22 days
ATGE_24	Wt	entire rosette after transition to flowering, but before bolting	23 days
ATGE_25	Wt	senescing leaves	35 days
ATGE_26	Wt	cauline leaves	21+ days
ATGE_27	Wt	stem, 2nd internode	21+ days
ATGE_28	Wt	1st node	21+ days
ATGE_29	Wt	shoot apex, inflorescence (after bolting)	21 days
ATGE_31	Wt	flowers stage 9	21+ days
ATGE_32	Wt	flowers stage 10/11	21+ days
ATGE_33	Wt	flowers stage 12	21+ days
ATGE_34	Wt	flowers stage 12, sepals	21+ days
ATGE_35	Wt	flowers stage 12, petals	21+ days
ATGE_36	Wt	flowers stage 12, stamens	21+ days
ATGE_37	Wt	flowers stage 12, carpels	21+ days
ATGE_39	Wt	flowers stage 15	21+ days
ATGE_40	Wt	flowers stage 15, pedicels	21+ days
ATGE_41	Wt	flowers stage 15, sepals	21+ days
ATGE_42	Wt	flowers stage 15, petals	21+ days
ATGE_43	Wt	flowers stage 15, stamen	21+ days
ATGE_45	Wt	flowers stage 15, carpels	21+ days
ATGE_46	clv3-7	shoot apex, inflorescence (after bolting)	21+ days
ATGE_47	lfy-12	shoot apex, inflorescence (after bolting)	21+ days
ATGE_48	ap1-15	shoot apex, inflorescence (after bolting)	21+ days
ATGE_49	ap2-6	shoot apex, inflorescence (after bolting)	21+ days
ATGE_50	ap3-6	shoot apex, inflorescence (after bolting)	21+ days
ATGE_51	ag-12	shoot apex, inflorescence (after bolting)	21+ days
ATGE_52	ufo-1	shoot apex, inflorescence (after bolting)	21+ days
ATGE_53	clv3-7	flower stage 12; multi-carpel gynoeceum; enlarged meristem; increased organ number	21+ days
ATGE_54	lfy-1	flower stage 12; shoot characteristics; most organs leaf-like	21+ days

(continued . . .)

**Sample ID**	**Genotype**	**Tissue**	**Age**
ATGE_55	ap1-15	flower stage 12; sepals replaced by leaf-like organs, petals mostly lacking, 2C flowers	21+ days
ATGE_56	ap2-6	flower stage 12; no sepals or petals	21+ days
ATGE_57	ap3-6	flower stage 12; no petals or stamens	21+ days
ATGE_58	ag-12	flower stage 12; no stamens or carpels	21+ days
ATGE_59	ufo-1	flower stage 12; filamentous organs in whorls two and three	21+ days
ATGE_73	Wt	mature pollen	6 wk
ATGE_76	Wt	siliques, w/ seeds stage 3; mid globular to early heart embryos	8 wk
ATGE_77	Wt	siliques, w/ seeds stage 4; early to late heart embryos	8 wk
ATGE_78	Wt	siliques, w/ seeds stage 5; late heart to mid torpedo embryos	8 wk
ATGE_79	Wt	seeds, stage 6, w/o siliques; mid to late torpedo embryos	8 wk
ATGE_81	Wt	seeds, stage 7, w/o siliques; late torpedo to early walking-stick embryos	8 wk
ATGE_82	Wt	seeds, stage 8, w/o siliques; walking-stick to early curled cotyledons embryos	8 wk
ATGE_83	Wt	seeds, stage 9, w/o siliques; curled cotyledons to early green cotyledons embryos	8 wk
ATGE_84	Wt	seeds, stage 10, w/o siliques; green cotyledons embryos	8 wk
ATGE_87	Wt	vegetative rosette	7 days
ATGE_89	Wt	vegetative rosette	14 days
ATGE_90	Wt	vegetative rosette	21 days
ATGE_91	Wt	leaf	15 days
ATGE_92	Wt	flower	28 days
ATGE_93	Wt	root	15 days
ATGE_94	Wt	root	8 days
ATGE_95	Wt	root	8 days
ATGE_96	Wt	seedling, green parts	8 days
ATGE_97	Wt	seedling, green parts	8 days
ATGE_98	Wt	root	21 days
ATGE_99	Wt	root	21 days
ATGE_100	Wt	seedling, green parts	21 days
ATGE_101	Wt	seedling, green parts	21 days

[a] For more details, see Schmid M. et al. *Nat. Genet.* 2005, **37**(5):501-506.
