# Supplementary material for: In plants, expression breadth and expression level distinctly and non-linearly correlate with gene structure
Source: Biol Direct. 2009 Nov 21;4:45. doi: 10.1186/1745-6150-4-45 (PMC2794262; doi:10.1186/1745-6150-4-45)
Supplement: Additional file 9 — Table S4.pdf. Sample information for rice microarray data. [file 1745-6150-4-45-S9.PDF]

## Table S4 - Sample information for rice microarray expression data.

| Sample | GEO accession numbers [a] |
|---|---|
| seedlings (7 days old) | GSM159259, GSM159260, GSM159261 |
| crown and growing point (ind.[b]) | GSM67052, GSM67053, GSM67054 |
| caryopsis (7 days after flowering) | GSM240994, GSM240995, GSM240996 |
| crown and growing point | GSM67058, GSM67059 |
| Inflorescence (10 - 15 cm) | GSM159198, GSM159199, GSM159200 |
| Inflorescence (15 - 22 cm) | GSM159201, GSM159202, GSM159203 |
| Inflorescence (22 - 30 cm) | GSM159204, GSM159205, GSM159206 |
| Inflorescence (3 - 5 cm) | GSM159192, GSM159193, GSM159194 |
| Inflorescence (5 - 10 cm) | GSM159195, GSM159196, GSM159197 |
| Mature leaf | GSM159180, GSM159181, GSM159182 |
| Leaf (75 days after germination) | GSM154957, GSM154958 |
| seedlings (11 days old) | GSM173086, GSM173080, GSM173089 |
| Root of 7-day-old seedlings | GSM159177, GSM159178, GSM159179 |
| Seed (0 - 2 days after pollination) | GSM159207, GSM159208, GSM159209 |
| Seed (11 - 20 days after pollination) | GSM159216, GSM159217, GSM159218 |
| Seed (21 - 29 days after pollination) | GSM159219, GSM159220, GSM159221 |
| Seed (3 - 4 days after pollination) | GSM159210, GSM159211, GSM159212 |
| Seed (5 - 10 days after pollination) | GSM159213, GSM159214, GSM159215 |
| Shoot apical meristem upto 0.5 mm at floral transition stage | GSM159186, GSM159187, GSM159188 |
| Stigma (before pollination) | GSM195218, GSM195219, GSM195220 |
| crown and growing point | GSM99870, GSM99871, GSM99872 |
| crown and growing point (ind.) | GSM99877, GSM99878, GSM99879 |
| crown and growing point | GSM99858, GSM99859, GSM99860 |
| crown and growing point (ind.) | GSM99864, GSM99865, GSM99866 |
| Leaf subtending the shoot apical meristem from mature plants | GSM159183, GSM159184, GSM159185 |
| Young inflorescence (upto 3 cm) | GSM159189, GSM159190, GSM159191 |
| Seed (5 days old) | GSM195230 |
| Mature anther | GSM195227 |
| Embryo (10 days after pollination) | GSM195228 |
| Endosperm (10 days after pollination) | GSM195229 |
| Ovary (before pollination) | GSM195221, GSM195222, GSM195223 |
| Coleoptiles (4 days old) | GSM159172, GSM159173 |
| Root (two-week old) | GSM195226 |
| Shoot (two-week old) | GSM195225 |
| Suspension cell (4-week cultured) | GSM195224 |

[a] more than one numbers indicate replicate experiments.
[b] ind. denotes Indica subspecies.
