# Supplementary material for: In plants, expression breadth and expression level distinctly and non-linearly correlate with gene structure
Source: Biol Direct. 2009 Nov 21;4:45. doi: 10.1186/1745-6150-4-45 (PMC2794262; doi:10.1186/1745-6150-4-45)
Supplement: Additional file 10 — Table S5.pdf. Correlation between expression pattern and sequence structural parameters for Arabidopsis genes. The expression data are the microarray data Ren et al. (2006) used in their study. For each structural parameter, ρs represent Spearman's rank sum corrleation coefficients between expression pattern and structural parameters, while partial ρs represent Spearman's partial correlations. Controlled variable for Expavg is expression width and that for Width is average expression level. Exptot, total expression level; Expavg, average expression level; Width, expression breadth. CDS, Coding Sequence; UTR, Untranslated Region. Level of significance: *, P > 0.05; **, 0.001 <P < 0.05; ***, 1e - 10 <P < 1e - 3; No asterisks indicates P < 1e - 10. Numbers in bold indicate highly significant partial correlations (P < 1e - 10). [file 1745-6150-4-45-S10.PDF]

Table S5 - Correlation between expression pattern and sequence structural parameters for *Arabidopsis* genes.

Parameters	Exp_{tot}	Exp_{avg}		$Width$	
	ρ	ρ	partial ρ	ρ	partial ρ
Length of primary transcript	0.194	-0.039	**-0.194**	0.248	**0.229**
Length of CDS	0.073	-0.136	**-0.180**	0.112	**0.119**
Number of introns	0.251	0.019	-0.162	0.296	**0.238**
Average exon length	-0.170	-0.077	**0.062**	-0.190	**-0.161**
Average intron length	0.252	0.139	0.009*	0.264	**0.127**
Total intron length	0.289	0.069	**-0.130**	0.330	**0.242**
5' UTR length	0.447	0.227	0.014*	0.465	**0.192**
3' UTR length	0.451	0.229	0.012*	0.467	**0.196**
5' intergenic length	-0.079	0.001	**0.128**	-0.104	**-0.179**
3' intergenic length	-0.140	-0.024	**0.072**	-0.159	**-0.124**

The expression data are the microarray data Ren et al. (2006) used in their study. For each structural parameter, ρs represent Spearman's rank sum correlation coefficients between expression pattern and structural parameters, while partial ρs represent Spearman's partial correlations. Controlled variable for Exp_{avg} is expression width and that for $Width$ is average expression level. Exp_{tot}, total expression level; Exp_{avg}, average expression level; $Width$, expression breadth. CDS, Coding Sequence; UTR, Untranslated Region. Level of significance: *, $P > 0.05$; **, $0.001 < P < 0.05$; ***, $1e-10 < P < 1e-3$; No asterisks indicates $P < 1e-10$. Numbers in bold indicate highly significant partial correlations ($P < 1e-10$).
