# Supplementary material for: In plants, expression breadth and expression level distinctly and non-linearly correlate with gene structure
Source: Biol Direct. 2009 Nov 21;4:45. doi: 10.1186/1745-6150-4-45 (PMC2794262; doi:10.1186/1745-6150-4-45)
Supplement: Additional file 11 — Table S6.pdf. The correlations between expression pattern and sequence structural parameters for Arabidopsis and rice genes. Genes were separately sorted according to their expression levels in each library; the ranks for each gene were then averaged to give the value of Expavg. Notably, in each library, a gene was taken as expressed only when > = 5 tags could be mapped onto it. For each structural parameter, the first line shows Spearman's rank sum corrleations with expression pattern, while the second line shows Spearman's partial correlations. Controlled variable for the columns of Expavg is expression width and that for the columns of Width is average expression level. CDS, Coding Sequence; UTR, Untranslated Region. Level of significance: *, P > 0.05; **, 0.001 <P < 0.05; ***, 1e - 10 <P < 1e - 3; No asterisks indicates P < 1e - 10. Numbers in bold indicate highly significant partial correlations (P < 1e - 10). [file 1745-6150-4-45-S11.PDF]

Table S6 - The correlations between expression pattern and sequence structural parameters for *Arabidopsis* and rice genes.

Parameters	Arabidopsis		Rice	
	Exp_{avg}	$Width$	Exp_{avg}	$Width$
Length of primary transcript	-0.019**	-0.001*	0.065	0.085
	**-0.194**	**0.193**	**-0.148**	**0.158**
Length of CDS	-0.183	-0.167	-0.094	-0.086
	**-0.178**	**0.161**	**-0.068**	**0.057**
Number of introns	0.107	0.120	0.091	0.101
	**-0.125**	**0.135**	**-0.076**	**0.088**
Average exon length	-0.157	-0.160	-0.056	-0.061
	0.021**	-0.036***	0.034***	-0.041
Average intron length	0.173	0.178	-0.014**	-0.007*
	**-0.043**	**0.060**	**-0.055**	**0.054**
Intron density	0.181	0.185	0.169	0.175
	**-0.059**	**0.077**	-0.036***	**0.057**
Total intron length	0.137	0.150	0.067	0.080
	**-0.124**	**0.137**	**-0.104**	**0.113**
5' UTR length	0.288	0.297	0.054	0.066
	**-0.083**	**0.112**	**-0.095**	**0.102**
3' UTR length	0.339	0.346	0.092	0.104
	**-0.064**	**0.099**	**-0.099**	**0.111**
5' intergenic length	-0.049	-0.054	0.021***	0.020**
	**0.052**	**-0.057**	0.010*	-0.007*
3' intergenic length	-0.069	-0.076	-0.058	-0.061
	**0.067**	**-0.074**	0.019**	-0.026***

Exp_{avg} was calculated as follows: genes were separately sorted according to their expression levels in each library; the ranks for each gene were then averaged to give the value of Exp_{avg}. Notably, in each library, a gene was taken as expressed only when ≥ 5 tags could be mapped onto it. For each structural parameter, the first line shows Spearman's rank sum corrleations with expression pattern, while the second line shows Spearman's partial correlations. Controlled variable for the columns of Exp_{avg} is expression width and that for the columns of $Width$ is average expression level. Intron density was calculated as the ratio of intron number to CDS length, i.e. intron number per coding base. CDS, Coding Sequence; UTR, Untranslated Region. Level of significance: *, $P > 0.05$; **, $0.001 < P < 0.05$; ***, $1e-10 < P < 1e-3$; No asterisks indicates $P < 1e-10$. Numbers in bold indicate highly significant partial correlations ($P < 1e-10$).
